# Eagle’s Syndrome Presenting as Peripheral Facial Palsy

**DOI:** 10.7759/cureus.22499

**Published:** 2022-02-22

**Authors:** Filipa Nunes, Maria João Fernandes, Mariana Silva, Beatriz Porteiro, Rita Dutschmann

**Affiliations:** 1 Internal Medicine, Hospital Professor Doutor Fernando da Fonseca, Lisbon, PRT; 2 Pneumology, Hospital Professor Doutor Fernando da Fonseca, Lisbon, PRT

**Keywords:** facial neuralgia, facial nerve, styloid process, eagle syndrome, peripheral facial palsy

## Abstract

Eagle’s syndrome (ES) is the elongation of the ossified styloid process that causes symptoms such as foreign body sensation, neck pain, and odynophagia. A styloid process greater than 25 mm in length should be considered abnormal. Facial palsy is a condition that affects the facial nerve and results in weakness or total paralysis of the facial muscles that control expression. Here, we describe a rare presentation of ES presenting as facial palsy. We present the case of a 62-year-old female who was admitted to the emergency department with right peripheral facial palsy. A computed tomography (CT) scan of the neck confirmed the diagnosis. The patient underwent conservative management and physical therapy, which resulted in good evolution with an improvement of symptoms. She was referred to the otorhinolaryngologist for surgical evaluation.

## Introduction

Eagle’s syndrome (ES) is the elongation of the ossified styloid process that causes symptoms such as foreign body sensation, neck pain, and odynophagia. A styloid process greater than 25 mm in length should be considered abnormal [1]. Facial palsy is a condition that affects the facial nerve and causes weakness or total paralysis of the facial muscles that control expression [2]. It can be divided into the following two types depending on the location of the insult: central facial palsy (due to supranuclear damage) and peripheral facial palsy (due to infranuclear damage). Bell’s palsy, whose cause is unknown although herpes simplex virus activation is considered to be the likely cause, is the most frequent cause of peripheral facial palsy. Other causes of peripheral facial palsy include trauma, particularly fractures of the petrous bone, Lyme disease, and complicated otitis media [2]. Compression of the facial nerve, infiltrations of the facial nerve by a tumor, and central lesions that involve the ipsilateral facial nerve nucleus or facial nerve tract in the pons are less frequent causes of peripheral facial palsy [2]. ES is a rare condition caused by an elongated styloid process that interferes with the function of the neighboring structures [3]. Here, we present the case of a 62-year-old female who was admitted to the emergency department with right peripheral facial palsy due to an ipsilateral enlarged styloid process.

## Case presentation

A 62-year-old female was admitted to the emergency department with a 24-hour evolution of right-sided facial weakness involving the mouth, eyes, and forehead, along with an incapacity to close her right eye and effacement of the right nasolabial fold. The taste sensation and salivation were intact. She did not have any history of headache, trauma, respiratory infection, ear symptoms, or any other paraesthesia or paralysis. She had a medical history of hypertension, dyslipidemia, and left styloidectomy.

On clinical examination, the patient had grade 3 hypertension, and her blood pressure was 200/95 mmHg. She had right facial nerve palsy grade 5 on the House-Brackmann facial paralysis scale. Other cranial nerve tests were normal. An evaluation of the ear, nose, and throat was performed by an otorhinolaryngologist and was normal. The patient did not have palpable lymph nodes or masses. The blood sample did not show any relevant alteration. A computed tomography (CT) scan of the head did not show acute ischemic or hemorrhagic lesions, and the head and neck CT angiography did not show any vascular involvement, such as compression, stenosis, dissection, or malformation in the angiographic evaluation. The vascular study was completed in the hospital, and the patient underwent magnetic resonance imaging (MRI) of the brain that excluded acute lesions.

**Figure 1:**
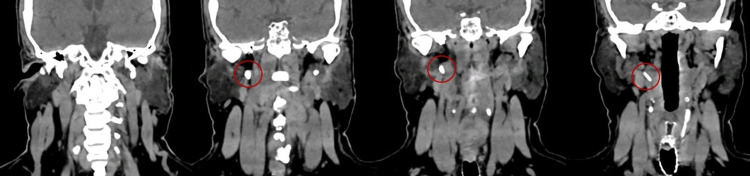
Elongation of the right styloid process (coronal plane).

**Figure 2:**
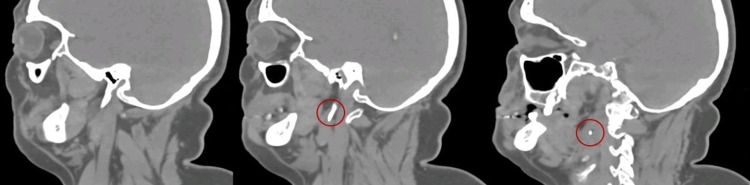
Elongation of the right styloid process (sagittal plane).

The patient was started on a five-day course of prednisolone 60 mg/day, then tapered 10v mg/day, for a total treatment time of 10 days. She also started physical therapy. During the follow-up consultation, her facial palsy had improved substantially with slight weakness of the facial muscle but normal symmetry of the face at rest. The patient still complained of foreign body sensation, odynophagia, and neck pain. A directed neck CT scan was performed to evaluate these symptoms. The neck CT scan revealed elongation of the right styloid process due to calcification of the stylohyoid ligament up to the lateral portion of the oropharynx, measuring 49 mm, and a shortened left styloid process due to previous surgery (Figures 1, 2).

The diagnosis of ES was made, and the patient was referred to the otorhinolaryngologist for surgical management.

## Discussion

Facial palsy due to ES is extremely rare. The incidence of ES differs in various studies [4]. An elongated styloid process is present in approximately 4% of the overall population, and, of these, only 4% present with symptoms caused by the elongation of the styloid process. Hence, the true incidence of ES is approximately 0.16%, with a female-to-male predominance of 3:1 [3]. The symptoms related to ES can be confused with facial neuralgias, migraine headaches, temporal arteritis, or temporomandibular joint disorder delaying the correct diagnosis and causing the patient to undergo unnecessary procedures and be prescribed superfluous drugs [4].

The diagnosis relies on physical examination and clinical history. In the oral examination, an elongated styloid process can be felt and induce pain [5]. Although plain skull radiographs may be sufficient to identify the anatomical elongation of the styloid process, CT of the head and neck, especially three-dimensional CT scan reconstruction is considered the gold standard for the assessment of the styloid process [5]. Three-dimensional CT scan reconstruction shows the complete details of the elongated styloid process and its relation to the adjacent structures. A lidocaine infiltration test in the tonsillar fossa can be performed, and, if symptoms resolve, it is most likely to be ES [4].

Facial palsy in this patient can be explicated by the compression of the extratemporal part of the facial nerve provoked by the elongated styloid process. Because the patient only had facial muscle weakness, the compression of the facial nerve is distal to the stylomastoid foramen, preserving taste, sublingual, submandibular, and lacrimal gland’s function, which would be affected if the damage of facial nerve was at a higher level [6].

The treatment is defined by the severity of symptoms. The first line of management is conservative, including non-steroidal anti-inflammatory drugs and analgesics to treat pain [4]. However, this conservative approach did not show satisfactory long-term results. In the case of failure of conservative management, the definitive treatment is surgical with styloidectomy [4]. Surgical styloidectomy in patients with a diagnosis of ES has a cure rate of 80% [7].

## Conclusions

ES can be misdiagnosed as its diagnosis and treatment are not simple. Its treatment is complex and best managed by a multidisciplinary team. Conservative treatment with painkillers can be used to alleviate pain. Surgery to remove the elongated styloid process is the definitive treatment and cures the majority of patients. In this case, the patient responded well to conservative management and similar treatment to that of peripheral facial palsy due to Bell’s palsy; however, based on the clinical features and imaging, ES was most likely the cause of her facial palsy.
